# Anatomy imaging and hemodynamics research on the cerebral vein and venous sinus among individuals without cranial sinus and jugular vein diseases

**DOI:** 10.3389/fnins.2022.999134

**Published:** 2022-09-27

**Authors:** Lu Liu, Yan Wu, Kaiyuan Zhang, Ran Meng, Jiangang Duan, Chen Zhou, Xunming Ji

**Affiliations:** ^1^Department of Neurology, Xuanwu Hospital of Capital Medical University, Beijing, China; ^2^Department of Emergency, Xuanwu Hospital of Capital Medical University, Beijing, China; ^3^Department of Radiology, Xuanwu Hospital of Capital Medical University, Beijing, China; ^4^Beijing Institute for Brain Disorders, Capital Medical University, Beijing, China; ^5^Department of Neurosurgery, Xuanwu Hospital of Capital Medical University, Beijing, China

**Keywords:** cerebral hemodynamics, cerebral vein, cerebral venous sinus, imaging, arachnoid granulations

## Abstract

In recent years, imaging technology has allowed the visualization of intracranial and extracranial vascular systems. However, compared with the cerebral arterial system, the relative lack of image information, individual differences in the anatomy of the cerebral veins and venous sinuses, and several unique structures often cause neurologists and radiologists to miss or over-diagnose. This increases the difficulty of the clinical diagnosis and treatment of cerebral venous system diseases. This review focuses on applying different imaging methods to the normal anatomical morphology of the cerebral venous system and special structural and physiological parameters, such as hemodynamics, in people without cranial sinus and jugular vein diseases and explores its clinical significance. We hope this study will reinforce the importance of studying the cerebral venous system anatomy and imaging data and will help diagnose and treat systemic diseases.

## Introduction

The cerebral venous system comprises superficial and deep veins, which contain nearly 70% of the brain’s blood volume and play a crucial role in maintaining normal cerebral perfusion. The cerebral veins converge in the intracranial venous sinus, and the patency of the venous sinuses is of great importance to ensure stable blood circulation and supply in the brain. Once blood stasis or thrombosis occurs, various clinical symptoms develop. Unfortunately, owing to the variation in cerebral venous anatomy and the lack of systematic imaging anatomical studies of cerebral veins and venous sinuses, it is difficult for patients with such diseases to obtain an accurate initial diagnosis, and they often miss the optimal time window for treatment.

At the end of the 20th century, Osborn (1999) obtained anatomical data on cerebral veins based on autopsies, which provided a basic understanding of cerebral vein distribution, structure, and classification. However, autopsies are difficult to perform widely in the population, and there are still many unanswered questions regarding the cerebral venous system. The development of modern imaging technology has allowed researchers to evaluate the cerebral veins in many individuals and make it possible to fully characterize the structure and function of the cerebral venous system. Multiple imaging techniques also provide perfect evidence for clinicians and researchers to deepen their understanding of human anatomy and explore systemic diseases’ causes, pathogenesis, and treatment.

In fact, in many cases, images interpreted as venous sinus thrombosis are simply due to the complete or partial absence of the dural sinus. Moreover, special structures, such as arachnoid granulations (AGs) or flow gaps, are sometimes closely related to the occurrence of venous sinus thrombosis, and clinicians and radiologists often find it difficult to distinguish or misdiagnose radiological images of these structures as pathological manifestations. For example, “delta signs” or “empty triangle” in upper sagittal sinus thrombosis can also occur in many other normal populations as a result of early bifurcation of the superior sagittal sinus (SSS) (before the occipital protuberance) and absence of “torcular herophili” (Zouaoui and Hidden, 1988), suggesting that only radiographic delta signs have no diagnostic value.

Existing pathological and imaging data on the venous sinuses are mainly derived from elderly patients and those with craniocerebral system diseases. Imaging data of the cerebral veins obtained from patients excluding such lesions are relatively limited. Therefore, an increasing number of researchers believe that it is meaningful to perform imaging studies of cerebral veins or cerebral venous sinuses in healthy people (excluding cranial sinus and jugular venous diseases) (Anand et al., 2022), which is conducive to understanding the normal anatomy of the cerebral venous system, excluding certain radiological images that are easily misdiagnosed as pathological, avoiding overdiagnosis, and providing baseline data to establish relevant diagnostic criteria.

In this study, we focused on and reviewed the anatomical variations, special structures, and hemodynamic manifestations of the cerebral venous system observed using several imaging techniques (ultrasound, computed tomography (CT), magnetic resonance imaging (MRI), and digital subtraction angiography [DSA]) in populations excluding the cranial sinus and jugular venous system diseases.

## Imaging techniques of cerebral veins

**FIGURE 1:**
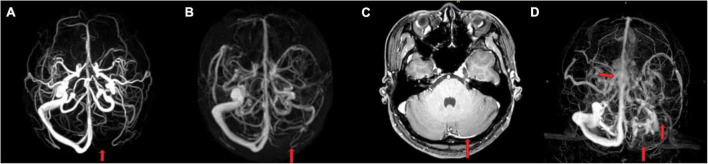
Comparison of the imaging findings between a 26-year-old healthy woman without cerebral venous system diseases and another clinical history **(A–C)** and a 26-year-old female cerebral sinus thrombosis patient **(D)**. **(A)** Phase-contrast-magnetic resonance venography (MRV) and **(B)** contrast-enhanced-MRV showed a suspicious aplastic or occlusive left transverse sinus (TS). While enhanced T1 image **(C)** showed that the left TS was not aplastic but slender. **(D)** The left TS, sigmoid sinus, and upper internal jugular vein were not visualized because of thrombosis, and the central part of superior sagittal sinus appeared rough and rugged.

**FIGURE 2:**
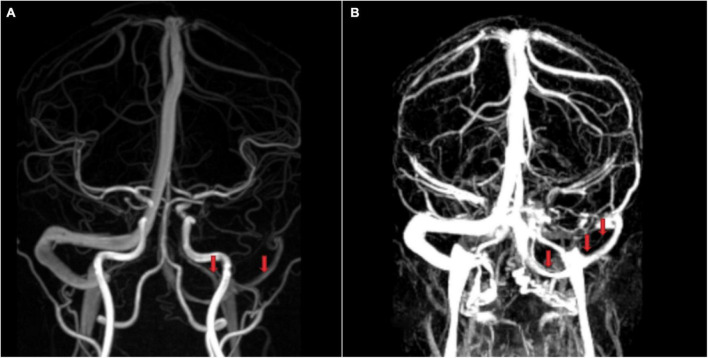
Images of the cerebral venous sinus from a 28-year healthy man without cerebral venous system diseases or another clinical history. **(A)** Phase-contrast-magnetic resonance venography (MRV) and **(B)** contrast-enhanced (CE)-MRV. CE-MRV provides better visualization of slender venous sinuses and superficial cerebral veins, which is highly significant for morphological classification.

Autopsy and radiology techniques are generally considered when there is a need to perform morphological descriptions of the cerebral venous anatomy or exclude cerebral venous system diseases. However, numerical data from autopsies often differ from those obtained *in vivo* using radiological techniques and cannot be used extensively in routine examinations. Radiological studies involved in this review mainly include DSA, CT venography (CTV), contrast-enhanced (CE)-magnetic resonance venography (MRV), time-of-flight (TOF) MR angiography (MRA), and phase-contrast (PC)-MRI. Commonly available imaging methods for evaluating the cerebral venous system have advantages and limitations (Figures 1, 2).

Digital subtraction angiography has long been considered the gold standard for diagnosing venous system diseases. However, it usually cannot describe the entire venous structure, only unilateral veins at a time (Waugh and Sacharias, 1992), and its X-ray exposure is still invasive, requiring contrast medium administration. These limitations limit their regular use (Paoletti et al., 2016). DSA is often unavailable when establishing imaging data of the cerebral venous system in healthy individuals.

Computed tomography venography is widely used in daily life and has better clinical applicability. However, for small veins that end at venous convergence, the resolution of this imaging method is often insufficient to observe them all (Brzegowy et al., 2021). CTV cannot be widely used in certain populations, such as children, patients with metallic vascular clips, or in the presence of highly enhanced tumors, due to high radiation doses (Rollins et al., 2005; Manara et al., 2010).

Magnetic resonance venography plays a role in studying the anatomy of the cerebral venous system and its variants, making it easier to distinguish its pathology from that of normal variants due to misinterpretation (Mattle et al., 1991; Ayanzen et al., 2000). The MRV does not involve ionizing radiation. It can show abnormal venous blood flow and intraluminal clot signals simultaneously, as well as some blood vessels close to the bone surface that are difficult to detect owing to artifacts on CT (Wetzel et al., 1999). Therefore, it is the first choice for non-invasive visualization of cerebral venous sinuses (Mattle et al., 1991). CE-MRV has become the preferred method among the different types of MRV for imaging the intracranial venous system, which can produce high-quality angiography but requires contrast agents. Therefore, it is not suitable for pregnant women or for individuals with renal insufficiency. TOF and PC-MRI, which are currently the most commonly used imaging technologies, do not require the use of contrast agents and have short imaging times (Liauw et al., 2000; Widjaja and Griffiths, 2004). Another velocity-selective (VS) MRA that can detect more small venous vessels than TOF MRA has also been developed, pending further evaluation for clinical application (Li et al., 2018). Compared to TOF MRV and CE-MRV, PC-MRV has a better background suppression effect on venous sinuses and large cortical veins. It can also quantify blood flow and demonstrate the direction of blood flow, which is essential for measuring the velocity of cerebral blood flow and observing the vein assessment of the cerebral venous sinus, internal jugular vein (IJV), and other intracerebral veins (Mehta et al., 2000; Bateman, 2003; Bateman et al., 2016; Rivera-Rivera et al., 2017), making it possible to assess the interaction between blood and cerebrospinal fluid flow in the cerebrospinal system. Many idiopathic diseases, such as hydrocephalus and intracranial hypertension, are associated with hemodynamic changes in intracerebral veins, and PC-MRI examination helps highlight the underlying blood flow and pulsatile disturbances in such patients (Lokossou et al., 2020), with the development of the 7 T research MR system, two dimensional (2D) PC-MRI was also used to acquire blood pulsation data from small cortical veins (0.6–2 mm) in healthy people (Driver et al., 2020). In summary, a simple and brief PC-MRV protocol may accurately investigate suspected structural or venous flow abnormalities in clinical settings, especially when contrast agent administration is unavailable in some patients (Jakhar et al., 2019).

Susceptibility-weighted imaging (SWI) mainly uses magnitude susceptibility or phase effects between venous blood and surrounding brain tissues (Haacke et al., 2004; Ong and Stuckey, 2010). Currently, SWI is widely used to visualize the cerebral venous system. It can describe both large (approximately 1 mm in diameter) and small (<1 mm in diameter) veins in the brain without the need for intravenous injection of contrast agents (Reichenbach et al., 1997) and is a reliable tool for measuring the inner diameter of the cerebral veins (Reichenbach et al., 2000; Reichenbach and Haacke, 2001; Cai et al., 2015). When detecting smaller venous structures and deep nuclei, SWI is significantly superior to MRV (Boeckh-Behrens et al., 2012) and DSA (Xia and Tan, 2013), and several studies have used this technique to explore the deep cerebral, cerebellar, and spinal veins (Ishizaka et al., 2010; Di Ieva et al., 2011; Sun et al., 2011; Chen et al., 2016).

In addition to studying venous anatomy using radiological techniques, new MRI techniques have gradually been developed to assess cerebral hemodynamic characteristics, such as dynamic susceptibility contrast imaging to reflect cerebral blood perfusion (Artzi et al., 2011; Leiva-Salinas et al., 2011) and blood-oxygen-level-dependent (BOLD) imaging to evaluate cerebrovascular reactivity and indirect blood oxygen under various physiological stimuli (Ogawa et al., 1990; Lu et al., 2009; Lee and Wehrli, 2020). Researchers have also achieved quantitative venous cerebral blood volume mapping using 3D MRI methods (Li et al., 2021). Velocity-selective excitation with arterial nulling, quantitative imaging of extraction of oxygen and tissue consumption, and T_2_-relaxation under spin tagging (TRUST) MRI were developed for the quantitative measurement of venous oxygenation (Lu and Ge, 2008; Bolar et al., 2011; Guo and Wong, 2012; Liu et al., 2016; Stout et al., 2018; Li et al., 2022).

The combination of these imaging methods applied to the cerebral venous system provides a large number of parameters that can provide a comprehensive description of the structural and functional characteristics of the cerebral venous system. The reference values for these parameters obtained from healthy subjects are of great significance.

## Imaging anatomy of the cerebral venous system

### Imaging anatomy of the cerebral venous sinus

Zouaoui et al. analyzed normal cerebral angiography of 100 subjects who had been excluded from any venous pathology, using a classification method that described four types of anatomic variation proposed by Edwards (1931) in an attempt to define the proportions of each type and compare them with previous studies (Zouaoui and Hidden, 1988). They found that the SSS ended at the midline in only 50.9 and 27.4% of the subjects had the SSS divided at the level of the internal occipital protuberance to form two lateral sinuses. However, in 23.5% of the subjects, the SSS was divided before reaching the internal occipital protuberance and did not form sinus confluence. The radiological results of this group could have been mistaken for thrombosis on the CT scans. Therefore, understanding the early forks of SSS is crucial (Zouaoui and Hidden, 1988).

In 2017, further analysis of the pattern for the division of SSS in 100 subjects using DSA also found that in most cases, SSS was divided into a sagittal plane (54%), followed by the parasagittal right (34%), and parasagittal left (12%). Interestingly, the presence of the parasagittal right division of the SSS was correlated with the right dominance of the transverse sinus (TS), straight sinus (SS), and IJV, while the presence of the parasagittal left division of the SSS was correlated with the left dominance of the TS, SS, and IJV, both of which were statistically significant. Epidemiological factors such as age and sex did not affect venous sinus drainage patterns in this cohort (Kitamura et al., 2017). Angiographic results in the venous phase in a study by Zouaoui et al. confirmed that the absence of the entire lateral sinus (transverse or sigmoid) is not equivalent to lateral sinus thrombosis, which may be due to embryonic hypoplasia and creates venous drainage dominance on the non-deleted side (Zouaoui and Hidden, 1988). Furthermore, Stoquart-Elsankari et al. (2009) also reported an interesting finding: six volunteers without left IJV flow collected the cerebral venous blood flow by contralateral IJV, and the TS flow was also significantly reduced (<20%) or null on the same side as the non-existent IJV flow. These morphological differences were closely related to the drainage pattern of the venous sinus. Using the venous phase of bilateral carotid angiography, Durgun et al. observed the venous sinus morphology and drainage pattern in 189 subjects without dural venous system lesions. They found that in most cases with symmetrical venous sinuses, the left and right TS had equal amounts of drainage, while in cases with right or left dominance, the drainage pattern of the sinus confluence appeared to be skewed toward the dominant side (Durgun et al., 1993). These drainage changes due to the anatomical morphology of the venous sinus should be considered when performing radical neck dissection and removing tumors that invade the IJV because ligation of the IJV on the dominant side in the presence of dominant drainage may cause venous infarction and other serious complications (Sugarbaker and Wiley, 1951).

In addition to using the gold standard technique of cerebral angiography to identify cerebral veins, many researchers have used non-invasive techniques such as MRV to conduct population studies. Goyal et al. retrospectively studied 1654 patients in India without congenital or acquired intracranial abnormalities, venous thrombosis, or previous surgery. Similar to previous studies, hypoplasia of the left TS was the most common anatomical variant (21.3%), hypoplasia of the right TS was relatively rare (5.5%), and aplastic/atretic was observed in 12 cases (0.7%). Only 1.6% had bilateral TS hypoplasia. The study by Goyal et al. was also the first to analyze the relationship between anatomical variations in the venous sinus and sex differences. In contrast, females had more symmetrical transverse and sigmoid sinus structures than males, while males were more likely to have left TS hypoplasia than females (24.9 vs. 19.3%; *P* = 0.009). The significance of the difference between sexes in lateral dominance has not yet been elucidated. Furthermore, SSS normally developed in 97.7% of subjects, and the most frequent anatomical variation of the SSS in the remaining population was atresia of anterior one-third SSS (0.9%), with no significant sex differences (Goyal et al., 2016). This proportion is slightly different from previous studies (1.8–9%) (Kaplan et al., 1972; Kaplan and Browder, 1973; Surendrababu et al., 2006; San Millán Ruíz et al., 2012).

Manara et al. prospectively analyzed coronal unenhanced 2D-TOF MRV images in 102 healthy individuals and evaluated the asymmetry and diameters of the TS. Most participants had TS asymmetry (71%), with the right TS significantly dominant (61%). It should be noted that the diameter measured in this study was not the actual anatomical diameter but represented the blood flow within the vessels. The goal is to provide possible measurement parameters for the clinical treatment of suspected thrombosis or the identification of anatomical variations. The average diameters of TS obtained by Manara et al. were 6.5 mm ± 1.84 on the right side and 5.1 mm ± 1.72 on the left side (*P* < 0.0001) (Manara et al., 2010), which were the same as the values of TS measured by Kitamura et al. using DSA (right, 6.4 mm vs. left, 5.4 mm) (Kitamura et al., 2017), but both were larger than those obtained by Baikov et al. The average diameters observed by them were 5.34 mm on the right side and 4.73 mm on the left (Baikov et al., 2007). All studies confirmed that the right TS had a larger diameter than the left TS, and no study in the cohort of Manara et al. had a right-left mean diameter <3 mm. The authors concluded that TS thrombosis or stenosis should be suspected when the mean right-left TS diameter approaches this value (Manara et al., 2010).

A recent retrospective study combined three-dimensional (3D) PC-MRV and CE-MRV to analyze the imaging data of 192 patients with normal MRI and without cerebrovascular disease manifestations. The proportions of TS asymmetry and left-side hypoplasia were similar to those reported by Goyal et al. while the most common variant in SSS, atresia of the anterior one-third, increased significantly (Goyal et al., 2016; Jakhar et al., 2019). This study confirmed that 3D PC-MRV is a good option for treating gadolinia allergy/renal insufficiency in pregnant patients, providing results comparable to those of CE-MRV. However, some chronic thrombi can manifest as linear filling defects and irregular outlines on CE-MRV, while the performance on PC-MRV is similar to that of sinus hypoplasia. Mohit et al. confirmed that multiplanar reconstruction and maximum intensity projection could be adjusted by adjusting the slice thickness to improve the accuracy of PC-MRV identification (Jakhar et al., 2019).

By reviewing the literature, we have summarized the trends in the anatomical morphology of the venous sinus (Table 1). Although some reported values were different, such as the average diameters of the TS obtained in several studies, we believe that this was due to the different inclusion criteria and measurement techniques (autopsy, DSA, CT, and MRV) used in various studies.

**TABLE 1:** Normal anatomy and variants of cerebral venous sinuses accessed by various imaging techniques.

Targeted cerebral venous sinuses	Enrolled participants	Age (year)	Imaging method	References
SSS	● 100 patients ● Without venous pathology	−	DSA	Zouaoui and Hidden, 1988
SSS, TS, SS, and IJVs	● 100 patients ● Without CVS diseases	56.3	DSA	Kitamura et al., 2017
TS, confluence of sinuses	● 189 cases ● Without any diagnosed CVS pathology	−	DSA	Durgun et al., 1993
SSS, TS, SS, and sigmoid sinus	● 1654 patients ● Without any congenital or acquired intracranial abnormality, CVST or previous surgery	37.98 ± 13.83	MRV	Goyal et al., 2016
TS	● 102 patients ● Without CVST or IIH	43	2D TOF MRV	Manara et al., 2010
SS, TS, and SSS	● 192 patients ● Without any manifestations of cerebrovascular diseases	0–85	3D PC-MRV and CE-MRV	Jakhar et al., 2019
SS, TS, SSS, IJVs, sigmoid sinus, and occipital sinus	● 100 patients ● Without ischemic changes, lacunar infarcts, tumors, myelination disorders, small vessel disease, other congenital and acquired morphological abnormalities, clinical indication of CVST and clinical suspicion of DVT	2–68	3D PC-MRV	Surendrababu et al., 2006

SSS, superior sagittal sinus; TS, transverse sinus; SS, straight sinus; IJV, internal jugular vein; CVS, cerebral venous system; CVST, cerebral venous sinus thrombosis; IIH, idiopathic intracranial hypertension; DVT, deep vein thrombosis; DSA, digital subtraction angiography; MRV, magnetic resonance venography; 2D, two-dimensional; 3D, three-dimensional; TOF, time-of-flight; PC, phase-contrast.

### Imaging anatomy of cerebral small veins

The cerebral venous system comprises superficial and deep veins. Previous studies have described the basic anatomy of the cerebral venous system; however, some small branching veins of the major veins have not been thoroughly studied. Diseases, such as stroke, leukoaraiosis, and developmental venous abnormalities are associated with these small veins. Compared to cerebral arteries, these small veins in the brain have received less attention. However, owing to the high inter-individual and intra-individual variability, it is easy to accidentally injure the veins adjacent to the area during surgery and can cause infarction or bleeding at the corresponding site. Therefore, evaluation of the anatomy of the cerebral small veins is critical for choosing an optimal surgical strategy (Table 2).

**TABLE 2:** Normal anatomy and variants of small cerebral veins accessed by various imaging techniques.

Targeted small veins	Enrolled participants	Age (year)	Imaging method	References
ICV and its primary tributaries	● 250 adults ● Without hydrocephalus, cerebral lesions, any injury that affects veins, significant imaging artifacts	52.3 ± 16.6	CTA	Brzegowy et al., 2019
Major veins in the pineal region	● 250 adults ● Without hydrocephalus, cerebral lesions, any injury that affects veins, significant imaging artifacts	19–89	CTA	Brzegowy et al., 2021
Venous network of brainstem	● 60 healthy adult volunteers ● Without any cerebral disease and its symptoms	20–28	SWI	Cai et al., 2015

ICV, internal cerebral vein; CTA, computed tomography angiography; SWI, susceptibility-weighted imaging.

Brzegowy et al. used CT angiography to retrospectively analyze the anatomy of internal cerebral veins (ICVs), Galen veins GV, basal veins BV, and other deep cerebral veins and their branches in 250 subjects, excluding cerebral parenchymal and vascular diseases, and attempted to establish a new classification for these veins (Brzegowy et al., 2019, 2021). The results showed that in 77% of the cerebral hemispheres, the thalamostriate vein was the only main tributary of the ICV, and this type was considered normal anatomy (Brzegowy et al., 2019). GV stenosis was observed in 51.2% of the study population. Surgical access through the GV might have been hindered in these patients. After classifying the BV according to the continuity of its trunk, they found that 66% of patients did not have the absence of the three segments of the BV trunk. The most common pattern was seen as a normal anatomical structure, while for the termination pattern of BV in the pineal region, they suggested that tributaries of the ICV and GV should be taken into account, as different BV termination patterns may imply different surgical approaches (Brzegowy et al., 2021).

Susceptibility-weighted imaging is preferred for showing the structure of small veins compared to CT, MRV (Boeckh-Behrens et al., 2012), and DSA (Xia and Tan, 2013). In recent years, some researchers have used SWI in healthy volunteers to observe the anatomy, variation, and drainage pattern of small veins in important areas, such as the third ventricle, brainstem, and pineal regions. For example, the anatomy and anatomic variations of the thalamostriate vein (Zhang et al., 2015) and anterior septal vein (Chen et al., 2016). Furthermore, evaluating the distance from the anterior septal vein-ICV junction to the foramen of Monro in healthy subjects provides a reference to avoid damage to the important nerve and vascular structures during surgery in the third ventricle area. SWI can also be used to quantitatively measure the diameter of these small veins, yielding a mean anterior septal vein diameter of 1.05–0.17 mm (range 0.9–1.6 mm) (Chen et al., 2016). The lateral mesencephalic vein was found in 75% of the subjects, and the mean outer diameter was 0.81 ± 0.2 mm (range, 0.5–1.2 mm) (Cai et al., 2015), which is similar to the values reported in anatomical studies (Matsushima et al., 1983; Türe et al., 1997).

### Imaging characteristics of flow gaps

Although the flow gap is not a clear-defined anatomical structure, it has been observed on CT or MR and has been described in several imaging studies of cerebral venous sinuses. Some researchers believe that the formation of the flow gap is mainly related to artifacts caused by slow intravascular blood flow, in-plane flow, and complex blood flow patterns or that there are problems with post-processing methods such as MR and angiography (Ayanzen et al., 2000). However, some studies have argued that the presence of AGs in cerebral venous sinuses could correspond to some extent, as their locations are often the same (Gailloud et al., 2001; Manara et al., 2010). The presence of flow gaps may increase the diagnostic difficulties in cases in which venous sinus thrombosis should be considered. Herein, we describe the imaging studies that involve flow gaps and AGs (Tables 3, 4).

**TABLE 3:** Imaging characteristics of flow gaps.

Most common location	Enrolled participants	Age (year)	Imaging method	References
L-FG: non-dominant side of TS O-FG: in the central and lateral part of TS	● 102 patients ● Without venous thrombosis or IIH	43	2D TOF MRV	Manara et al., 2010
Non-dominant side of TS	● 100 patients ● Without any intracranial abnormality, congenital anomaly, venous thrombosis, tumor, or previous craniotomy	0–83	2D TOF MRV	Ayanzen et al., 2000
Middle part and the dominant side of TS	● 111 patients ● Without MR evidence of structural brain lesions, hydrocephalus, or abnormalities of CSF composition, drug intake, or systemic disease	16–68	3D PC-MRV	Bono et al., 2003
At the junction of sigmoid sinus and TS or the outermost part of TS	● 100 patients ● Without ischemic changes, lacunar infarcts, tumors, myelination disorders, small vessel disease, other congenital and acquired morphological abnormalities, a clinical indication of CVST, and clinical suspicion of DVT	2–68	3D PC-MRV	Surendrababu et al., 2006

L-FG, linear flow gap; O-FG, oval flow gap; TS, transverse sinus; IIH, idiopathic intracranial hypertension; 2D, two-dimensional; 3D, three-dimensional; MRV, magnetic resonance venography; TOF, time-of-flight; PC, phase-contrast; CSF, cerebrospinal fluid; CVST, cerebral venous sinus thrombosis; DVT, deep vein thrombosis.

**TABLE 4:** Characteristics of arachnoid granulations accessed by various imaging techniques.

Imaging Feature	Most common location	Enrolled participants	Age (year)	Imaging method	References
Isodensity or low density with clear boundaries	TS and directly connected to adjacent veins	● 573 patients ● Without dural sinus thrombosis, traumatic or surgical sinus disruption	−	CE-CT, 2D TOF MRV	Leach et al., 1996
–	At the junction of the vein of Labbé and TS	● 57 patients ● Without hemorrhage and no angiographically proven aneurysm, or other intracranial hemorrhage	16–87	DSA	Gailloud et al., 2001
Round or oval with uniform internal density	SSS and adjacent cortical veins	● 110 patients ● Without dural sinus thrombosis, brain tumor or AVM	0–76	CE 3D turbo-flash MRV	Haroun et al., 2007
T_1:_Hypointense signal T_2_: similar signals to CSF T_2_ Flair: never showed high signals	TS	● 1118 patients ● Without dural sinus thrombosis, traumatic or neoplastic disease involving the dural sinuses	0–93	MRI (T_1_, T_2_, FLAIR)	Ikushima et al., 1999

TS, transverse sinus; CT, computed tomography; 2D, two-dimensional; 3D, three-dimensional; MRV, magnetic resonance venography; TOF, time-of-flight; PC, phase-contrast; CE, contrast-enhanced; DSA, digital subtraction angiography; SSS, superior sagittal sinus; AVM, arteriovenous malformation; CSF, cerebrospinal fluid; MRI, magnetic resonance imaging.

Alper et al. described the presence of flow gaps in the TS in 24% of the normal population (Alper et al., 2004). Ayanzen et al. observed the venous sinuses of 100 subjects with normal MRI using 2D-TOF MRV and found that the proportion of TS with flow gaps was as high as 31 and 90% of the flow gaps appeared on the non-dominant side. However, almost no flow gaps were observed in the normally dominant TS, SSS, SS, and GV. Consequently, it is believed that the potential pathology should be considered if there are blood flow gaps in these parts (Ayanzen et al., 2000).

Manara et al. also used 2D-TOF MRV to divide blood flow gaps by shape (oval flow gap [O-FG] and linear flow gap [L-FG]) and described their position and incidence, respectively. They found that L-FG in normal people appeared on the non-dominant side of the TS, and two-thirds of L-FG did not involve the distal end of the TS, while O-FG was mostly located in the central and lateral parts of the TS. Therefore, if L-FG appears on the dominant side of the TS, particularly on the distal side, and O-FG appears in the middle segment of the TS, sinus thrombosis should be considered (Manara et al., 2010).

Bono et al. defined flow gaps when the lack of signal flow in the MRV was less than or equal to one-third of the sinus length. Unlike the studies by Ayanzen et al. (2000) and Manara et al. (2010), they also observed flow gaps in 36.4% of the dominant TS, which they attributed to differences caused by 3D PC-MRV. In the study by Bono et al. (2003) only 1.8% of the subjects developed flow gaps in bilateral TS, and almost all remaining flow gaps were only on one side, suggesting that bilateral blood flow disturbances in TS rarely occur in individuals with normal cerebrospinal fluid (CSF) pressure. Bono et al. (2003) also found that 85% of the flow gaps appeared in the middle part of the TS, whereas the flow gaps studied by Surendrababu et al. were mainly located at the junction of the sigmoid sinus and the TS or the outermost part of the TS. Interestingly, Surendrababu et al. (2006) did not observe flow gaps in the dominant transverse and sigmoid sinuses, even though the technique used was the same as that used by Bono et al.

It should be noted that although it is controversial whether flow gaps exist in the dominant venous sinus of normal subjects due to differences in technique and study design, flow gaps in these studies were consistent with some AGs that are common in the lateral TS or venous entrance sites^[55]^. This suggests that flow gaps may be generated due to protruding AGs or the corresponding lumen damage caused by thrombosis formed based on these anatomical structures, resulting in a pressure gradient or interference with blood flow, whereas only a few flow gaps in the proximal TS may be explained by proximal TS hypoplasia (Bono et al., 2003).

### Imaging characteristics of arachnoid granulations

**FIGURE 3:**
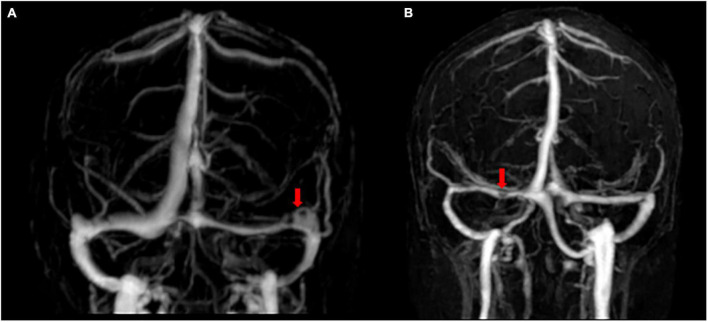
Flow gaps and arachnoid granulations in cerebral venous sinus using contrast-enhanced-magnetic resonance venography. **(A)** Two flow gaps are on the non-dominant side and at the junction of the sigmoid sinus and the transverse sinus (TS). **(B)** A small flow gap is on the proximal TS. After evaluation by radiologists, these flow gaps were considered to be arachnoid granules.

Arachnoid granulations are focal arachnoid eminences that usually enter the sinus lumen or lacunae lateralis (Potts et al., 1972). As a well-known normal anatomical structure (Figure 3), the AG was originally derived from the autopsy of patients, suggesting that it appears most often in the superior sagittal region (Mamourian and Towfighi, 1995; Haybaeck et al., 2008), whereas subsequent observations with CT or MRI indicated that AGs are often present at the junction of the TS and some veins (Leach et al., 1996; Ikushima et al., 1999; Gailloud et al., 2001; Haroun et al., 2007).

There are few reports of AGs, most of which are case reports. Roche and Warner suggested that the presence of AGs is also common in the dural sinuses of healthy individuals and is often unrelated to associated symptoms, as the presence of AGs reported in most data does not cause severe pathological changes or clinical symptomatology (Roche and Warner, 1996). However, it has also been suggested that larger AGs may create relative lumen compromise, leading to pressure gradients or disturbed flow. This can lead to venous hypertension (if the SSS or dominant TS is involved) or thrombosis (if the blood flow is sufficiently slow or is in a hypercoagulable state). Therefore, from a clinical point of view, it is necessary to clarify the AGs presented by different imaging techniques in healthy individuals to distinguish them from sinus thrombosis, meningioma, cavernous hemangioma, and meningocele.

Several imaging studies conducted on healthy individuals are committed to determining the criteria that can characterize AGs, such as their size, shape, number, and location. For example, after confirming that the brain MRI and MRV of the subjects were normal and excluded cerebral venous sinus thrombosis (CVST), brain injury, brain tumor, and other diseases, Leather et al. found that 24% of 573 patients accessing CE brain CT images had AGs in the venous sinus (Leach et al., 1996), which was similar to the 21.1% reported by Gailloud et al. (2001) using DSA. AGs observed by Leach were mainly located in the TS and presented as isodensity or low-density with clear boundaries (Leach et al., 1996). A total of 83% of the AGs were round or oval with uniform internal density (Haroun et al., 2007), which could be differentiated from the high density of acute thrombi shown on CT. Furthermore, Leather et al. also found that AGs present in 13% of 100 MRI cases showed hypointense signals on T_1_ and flow empty signals at the proximal and distal ends (Leach et al., 1996). Another study of 1118 patients with non-CE conventional MRI found that 10.0% of the dural sinuses had AGs and that only showed signals similar to CSF on T_2_-weighted images, which were helpful for the identification of dural sinus thrombosis and meningioma, but not applicable for epidermoid cysts, hemangiomas, and other lesions that have signals similar to those of the CSF on both T_1_- and T_2_-weighted images. Ikushima found that AGs did not show high signals in T_2_ FLAIR images, whereas epidermoid cysts showed mild-to-moderate high signals. Therefore, the FLAIR sequence can distinguish between AGs and epidermoid cysts (Ikushima et al., 1999).

Furthermore, some special positional relationships between AGs and venous entrances have been confirmed in previous imaging studies. Leather et al. observed that AGs were directly connected to adjacent veins in 62% of cases on CT and 85% of cases on MR (Leach et al., 1996), and Gailloud found that AGs were present at the junction of the vein of Labbé and the TS in 57 angiograms (Gailloud et al., 2001). Using CE 3D turbo-flash MRV, Haroun et al. found that 75% of AGs were associated with cortical veins (Haroun et al., 2007); all of the above can be explained by the fact that AGs are intraluminal protrusions of the venous layer formed where the perivascular pia maters penetrate the sinus dura (Krisch, 1988). Consequently, when there is a filling defect of the venous sinus connecting to a corresponding unobstructed vein on imaging, the possibility of AGs should be considered rather than simply suspecting venous sinus thrombosis. Furthermore, imaging studies also found that individuals with AGs were slightly older than those without AGs and that AGs tended to increase in diameter with age. No sex advantage was found in these studies (Leach et al., 1996; Ikushima et al., 1999; Gailloud et al., 2001; Haroun et al., 2007). However, owing to different inclusion criteria, the age at which AGs are predisposed varies according to different imaging studies, and whether AG changes in shape and function with age requires further verification by imaging techniques in large sample populations.

## Imaging characteristics of cerebral venous hemodynamics

Monro–Kellie considered the skull as a rigid closed box characterized by dynamic interactions between the brain tissue, arterial blood, venous blood, and CSF (Greitz et al., 1992). Many previous studies have emphasized that cerebral artery contraction and expansion play vital roles in regulating normal intracranial pressure and CSF circulation. However, owing to the complexity of the cerebral venous system structure and individual differences, the actual role and mechanism of the cerebral venous system in intracranial and extracranial hemodynamic changes remain controversial, and researchers have used different imaging techniques to study them (Tables 5, 6).

**TABLE 5:** Cerebral venous hemodynamics accessible by various imaging techniques.

Targeted objects	Venous blood flow	Venous velocity	Venous pulsatility	Enrolled participants	Imaging method	References
SSS, SS, TS	+	−	+	● 18 HYVs ● Without any neurologic, psychiatric, or severe general disease, alcoholism, or abnormalities detected with clinical MRI exams	PC-MRI	Stoquart-Elsankari et al., 2009
IJVs, SS, sagittal sinuses	+	−	+	● 19 HEVs and 16 HYVs ● Without cognitive decline, cerebral neurological diseases, or relevant cerebrovascular risk factors	PC-MRI	Lokossou et al., 2020
Small cortical veins	−	−	+	● 8 healthy volunteers	PC-MRI at 7T	Driver et al., 2020
SSS, SS, IJVs	+	−	−	● 26 healthy volunteers ● Without cardiovascular, cerebrovascular, kidney, neurological diseases, cardiovascular-acting medications, smoking currently or in the past	PC-MRI	Tarumi et al., 2021
TS	−	+	+	● 43 healthy term neonates ● Without maternal illnesses, vacuum or forceps deliveries.	Doppler ultrasound	Baytur et al., 2004
SSS	+	+		● 14 healthy volunteers ● Without history of neurologic diseases	MR velocity mapping	Gideon et al., 1996
SSS, TS	+	+	−	● 15 healthy young volunteers ● Without brain abnormalities	PC-MRI	Mehta et al., 2000

SSS, superior sagittal sinus; TS, transverse sinus; SS, straight sinus; HYVs, healthy young volunteers; IJV, internal jugular vein; HEVs, healthy elderly volunteers; PC, phase-contrast; MRI, magnetic resonance imaging.

**TABLE 6:** Posture-induced imaging changes in cerebral veins and venous sinuses.

Related veins	Enrolled participants	Age (year)	Imaging method	References
IJVs	10 healthy volunteers	29 ± 7	PC-MRI	Alperin et al., 2005a
IJVs and the cervical vertebral plexus	5 healthy volunteers	32–47	MRI	Niggemann et al., 2011
Cortical vein, PCV, SMCV ICV, SSS, SS, TS, sigmoid sinus, SPS, IPS	20 healthy volunteers	30–55	Upright head CTA	Kosugi et al., 2020
IJVs and vertebral veins	23 healthy young adults	25	Color-coded duplex sonography	Valdueza et al., 2000

IJV, internal jugular vein; PCV, precentral cerebellar vein; SMCV, superficial middle cerebral vein; PC, phase-contrast; MRI, magnetic resonance imaging; SSS, superior sagittal sinus; TS, transverse sinus; SS, straight sinus; SPS, superior petrosal sinus; IPS, inferior petrosal sinus; CTA, computed tomography angiography.

### Venous blood flow

Understanding the normal blood volume in different veins or sinuses is important. Studies have shown that abnormal deviations in venous blood flow can be used to predict disease or to guide treatment. For example, venous flow patterns have been used to predict the likelihood of venous stroke in patients with venous thrombosis and to evaluate stenting in patients with hydrocephalus and venous stenosis with significant hemodynamic changes (Levitt et al., 2016; Schuchardt et al., 2017). Moreover, venous flow measurements can help clarify hydrocephalus (Rasulo et al., 2017), and regional venous flow measurements may help to understand the pathophysiology of traumatic brain injury (Doshi et al., 2015).

Jill B et al. measured venous blood flow in the SSS, SS, and IJV using PC-MRI and found that SSS, SS, and IJV accounted for 46 ± 9, 16 ± 4, and 79 ± 1% of arterial inflow, respectively. It can be seen that venous blood from the cerebral cortex (i.e., flowed through SSS) and venous blood from deep brain structures (i.e., flowed through SS) drain around 62% of the total cerebral blood (i.e., total artery inflow). Since IJV comprises approximately 79% of the total cerebral blood flow, 17% is drained through the cerebral and cerebellar veins, which drain into the venous sinuses after venous sinus fusion (e.g., Labbé vein). Furthermore, approximately 21% of cerebral blood flow is not drained by IJVs but by the venous plexus, which subsequently flows into the cervical and external jugular veins (De Vis et al., 2018). However, the distribution of cerebral blood flow did not change significantly in the state of hypercapnia; some blood flow may drain through the lymphatic system surrounding the sinus veins (Louveau et al., 2015). This study demonstrated the main drainage venous blood flow and proposed a method to calculate cerebral venous and venous sinus flow using PC-MRI that integrates the region of interest of the vessel obtained from multiple repetitions. This allows patients to achieve non-invasive and rapid measurements of relative blood flow in all cerebral veins and venous sinuses while undergoing diagnostic MRI.

Lokossou et al. used PC-MRI to measure intracranial and extracranial venous blood flow in two groups of healthy people (younger and older), with SS and SSS representing intracranial venous blood flow, and jugular veins representing extracranial cerebral venous blood flow. Interestingly, the measured values of extracranial and intracranial venous cerebral blood flow were similar in the younger group (449 ± 173 vs. 478 ± 94 ml/min), while, in the elderly group, cerebral blood flow through the straight and superior sagittal sinuses was 29% lower than that through the jugular vein (Lokossou et al., 2020), which also consistent with the findings of Jill et al., indicating that the contribution of the extracerebral veins, in addition to the cerebral sinuses and peripheral venous drainage system, cannot be ignored.

Rhythmic handgrip (RHG) is a typical characteristic of exercise-induced cerebral and cardiovascular hemodynamic responses. Tarumi et al. investigated the impact of moderate-intensity RHG exercise on cerebral blood flow (CBF) and CSF flow dynamics in young healthy adults. RHG significantly reduced the blood stroke volume (SV) of the IJV, SSS, and SS compared with the resting control group, which was associated with increased cerebrovascular resistance and may be explained by the Monro–Kellie hypothesis. Although blood flow rates in SSS and SS remained unchanged during RHG, the blood flow rate in IJV increased as in the internal cerebral artery (ICA), vertebral artery (VA), and other arteries, which may be due to greater blood SV reductions in these sinuses (16–20%) than in the ICA, VA, and IJV (9–12%). Anatomical differences between the venous sinus, arteries, and veins are also one of the reasons for the above results. The SSS and SS are covered by a thick dura mater, whereas the walls of the arteries and veins are more compliant. Such structural differences in the sinus wall may lead to different regional blood flow distributions under dynamic conditions. Furthermore, since the IJV is a major pathway for venous drainage, the correlation analysis in this study suggested that increased heart and respiratory rates during RHG may also increase IJV flow (Tarumi et al., 2021). These findings showed that the improvement in brain health by exercise and its preventive effect against cerebrovascular and nerve dysfunction could be associated not only with changes in arterial CBF but also with CSF and venous hemodynamic alternation, which has potential clinical implications for cerebrovascular and neurological diseases. After all, in the current literature, most studies examining the effect of exercise on cerebral hemodynamics have focused on arterial CBF (Smith and Ainslie, 2017).

### Venous velocity

Baytur et al. believed that due to the superficial course under the skull and the different wall structures of the fetal venous sinuses, they may be more sensitive to perinatal events, such as increased intracranial pressure and blood gas changes, and compared the perinatal fetal cerebral veins and arteries examined by Doppler ultrasound. In contrast to cerebral arterial blood velocity, which decreased significantly 1 h after birth, cerebral venous blood velocity remained constant after birth in infants born vaginally or by cesarean section, indicating that the regulation of venous blood flow may differ from that of the arterial system, and is likely to regulate arterial blood flow in a different and more complex manner (Baytur et al., 2004).

A study of MR velocity mapping by Gidenon et al. in a healthy adult population did not show a significant correlation between mean velocity in the SSS and age; however, there was a tendency toward a reduction in mean blood flow with increasing age, perhaps because the SSS area decreased with age, and whether the venous sinuses adapt to the CBF similar to other cerebral vessels remains an open question (Gideon et al., 1996).

Further experiments were conducted to investigate the effects of physiological stressors on dural sinus blood flow, such as linking cerebrovascular circulation with the cardiovascular system, by comparing the effects of the Valsalva maneuver, which generates positive intrathoracic pressure, and Müeller’s maneuver, which generates negative intrathoracic pressure, on brain-heart circulation in PC-MRV images. They found that deep inspiratory breath-holding and deep expiratory breath-holding may regulate intrathoracic pressure, right atrium/left ventricular filling, and reduce venous return to the heart, resulting in decreased dural sinus flow and velocity (Mehta et al., 2000).

### Venous pulsatility

Some researchers believe that cerebral veins are collapsible and can passively change their cross-sectional configuration in response to applied pressure and that their pulsations are mainly copies of the arterial (Schaller, 2004).

Stoquart calculated the venous pulsatility index (VPI) at both the cervical and cerebral levels after analyzing PC-MRI images in healthy volunteers and found that the extracranial compartment, represented mainly by the jugular veins, has a higher elasticity and therefore a higher VPI than the SSS, which can represent the higher pulsatility and improved indirect compliance of the venous intracranial or cervical compartments (IJV or EV), thus playing a role in the regulation of intracranial pressure (Stoquart-Elsankari et al., 2009), also indicating that the functions of the intracranial and extracranial venous drainage systems are different.

Some authors have used PC-MRI and have found an increase in cerebral arterial pulsatility with age (Wåhlin et al., 2014; Zarrinkoob et al., 2016). To determine whether venous pulsation also has age-related changes, Lokossou et al. used PC-MRI to analyze venous flow pulsations in healthy younger (mean age, 31 ± 7 years) and elderly (mean age, 73 ± 6 years) groups. Venous blood pulsatility in elderly people is higher than in younger people, suggesting that cerebral hemodynamic parameters are related to age to some extent (Lokossou et al., 2020). Intracranial VPI in both groups was significantly lower than extracranial VPI (HYV: 0.25 ± 0.16 vs. 0.47 ± 0.25; HEV: 0.52 ± 0.16 vs. 0.86 ± 0.40), as Stoquart-Elsankari et al. (2009) reported previously. Venous blood flow pulsatility was lower in the intracranial compartment than in the extracranial compartment irrespective of age.

Driver et al. used PC-MRI at 7 T in eight healthy participants and defined a statistical parameter, PCNR, to characterize whether a waveform was pulsatile. They demonstrated the presence of flow pulsatility in small cerebral cortical veins (0.6–2 mm) for the first time and revealed significant pulsatility in 116 of the 146 veins (79%) assessed in the parietal and frontal regions, determined by PCNR > 3.9 (Driver et al., 2020). Because PCNR is present in all veins, it can also be used to show the distribution of pulsatility in cortical veins in healthy brains, even in patients in the future, which is of great significance for studies of cerebral venous function and provides the potential for a more detailed investigation of cerebral hemodynamics, intracranial pressure, and cerebral compliance.

### Posture-related changes

Postural changes strongly affect intracranial fluid dynamics and cerebral hemodynamics, including intracranial compliance and pressure (Feldman et al., 1992). In recent years, the effect of changes in body posture on cerebral venous outflow has attracted the attention of researchers. In a 2000 Lancet study using ultrasound in cerebral venous drainage, the jugular vein was the predominant drainage route in the supine position, whereas the vertebral vein system was the main outflow pathway in the upright position (Valdueza et al., 2000). Alperin et al. subsequently used MRI technology to make a further quantitative comparison of parameters, such as venous drainage and venous pulsation during postural changes, and confirmed that blood flow primarily passed through the IJVs (approximately 50%) in the supine position, which was not completely consistent with previous results of approximately 90% reduction (Valdueza et al., 2000), and The blood flow velocity also increased, while in the upright position, the IJVs collapsed and drained through secondary veins. They also found that the amplitude of the arterial waveform was similar in both the supine and sitting positions; however, the amplitude of the venous flow was significantly reduced in the upright posture, suggesting that the venous flow was much less pulsatile. A possible explanation for this is that gravity preferentially redistributes blood volume to the lower part of the body in the upright state, which reduces the blood volume of the cerebrovascular system and then increases cerebrovascular compliance, which is greatly affected by cerebrovascular compliance, leading to a decrease in pulsating venous outflow (Alperin et al., 2005a,b).

The main reason for the collapse of IJVs is the negative pressure of thin-walled IJVs in the upright position because of their location above the heart (San Millan Ruiz et al., 2002; Gisolf et al., 2004). The vertebral venous velocity increased significantly at this time. Niggemann et al. (2011) also quantified IJV with positional MRI in a later study, confirming again that its cross-sectional area changed from 100 mm^2^ ± 29 mm^2^ in the supine position to 11 mm^2^ ± 2 mm^2^ in the upright position.

These differences can be explained by differences in the distribution of venous drainage between subjects, and better quantification and accuracy of drainage parameters using MRI. For radiologists or spinal surgeons, IJV collapse images obtained in this upright state must be considered pitfalls of positional MRI rather than lesions, and imaging differences in venous drainage caused by postural changes are difficult to distinguish based on pathology. However, clinical issues such as bilateral neck dissection, metastatic spread of tumors, and controversies regarding head positioning during elevated intracranial pressure have emphasized the importance of postural influence on cerebral venous drainage. Continuous research using advanced imaging techniques helps answer these questions. For example, using upright head CT angiography, Kosugi et al. also found that when healthy subjects moved from the supine position to the upright position, the venous channels, including IJVs and external jugular veins, collapsed, diverting the main cerebral venous drainage from the internal jugular system to the vertebral venous system, while the intracranial vessels, including the arteries, veins, and venous sinuses, did not show significant postural changes compared to the venous structures at the craniocervical junction (Kosugi et al., 2020). Interestingly, they speculated that in patients complaining of orthostatic headache and suspected CSF hypovolemia; upright CT may reveal dilated intracranial venous structures compared to the supine position, as venous sinuses have been shown to expand to compensate for the loss of intracranial cerebrospinal fluid volume in patients with low intracranial pressure (Farb et al., 2007), although the sensitivity of upright head CT angiography for the detection of such diseases requires further study.

## Conclusion and perspectives

Imaging is a common auxiliary tool used by neurologists to diagnose various diseases. In contrast to cerebral arterial system diseases, such as ischemic stroke, it is often difficult to obtain effective imaging data in time to diagnose and treat diseases of the cerebral venous system. There are several possible reasons for this finding. First, the anatomy of the cerebral venous system itself has more morphological variations and individual differences, whereas many clinicians and radiologists have a poor understanding of the anatomy and corresponding imaging of the cerebral venous system. Second, the onset of cerebral venous system disease is often insidious and affects patients’ daily lives. Since symptoms are changeable at the time of onset, patients often choose symptom-related departments rather than consulting neurologists for the initial diagnosis, resulting in varying degrees of delay in diagnosis and treatment. This review compares the advantages and disadvantages of different imaging techniques for displaying the structure and function of the cerebral veins (Table 7). DSA is the gold standard for venous diseases but is invasive, and CTV involves radiation exposure. CE-MRV is easy to perform in the clinic but also has corresponding contraindications since it requires contrast media administration, and special imaging techniques such as bold MRI can detect specific cerebral venous blood flow dynamics parameters, while there is no more evidence to support its use in venous diseases. In particular, we summarize a series of imaging studies of the cerebral venous system carried out in people, excluding those with cranial sinus and jugular vein diseases. Current research in this area has obtained data on the anatomy of the cerebral veins and venous sinuses, special structures such as AGs, and cerebral venous hemodynamic parameters under normal physiological conditions. Although the results and conclusions of some studies are not completely consistent due to the different imaging methods and inclusion standards, these data from “relatively healthy volunteers” compared to those with cerebral venous system lesions also provide valuable information to clarify the structure and function of the cerebral veins and venous sinuses under normal physiological conditions, especially some basic parameters that may be used as future baselines for the identification of system diseases.

**TABLE 7:** Summary of advantages, clinical limitations, and application recommendations of usual imaging techniques for cerebral venous system.

Imaging technique	Advantages	Clinical limitations	Recommendations for clinical application
DSA	● Visualization of blood vessels *in vivo* in a bony or dense soft-tissue environment clearly	◆ Radiation exposure ◆ Not for patients with pregnancy, contrast material allergy or abnormal renal function ◆ Depicting unilateral venous structures	➣ Venous pressure measurements ➣ Venous diameters measurements ➣ The gold standard for venous diseases such as cerebral sinus thrombosis (CVT) ➣ Excluding other causes of bleeding, such as a distal aneurysm or dural arteriovenous fistula ➣ Endovascular procedures such as thrombectomy ➣ Clarifying equivocal CT/MRI findings
CTV	● Quicker to perform and interpret ● Less movement artifact ● Visualization of bony grooves	◆ Radiation exposure ◆ Not for patients with pregnancy, contrast material allergy or abnormal renal function ◆ Reducing diagnostic accuracy as its background bone suppression ◆ Limited diagnostic value for diagnosing cortical vein thrombosis	➣ Detailed anatomic images of the deep and superficial cerebral veins ➣ A reliable alternative to DSA for the diagnosis of CVT ➣ Suitable for comatose or uncooperative post-seizure patients
TOF MRV	● Sensitive to slow flow ● No use of contrast materials ● Shorter acquisition times (5–8 min)	◆ Cause artificial flow gaps ◆ Not sensitive for assessing smaller veins ◆ Limited diagnostic value for diagnosing cortical vein thrombosis	➣ Highly reliable for CVT in larger cerebral veins and sinuses ➣ The coronal sequence is valuable and should be interpreted in conjunction with the other sequences ➣ Evaluating pathology in the vessels which are close to the bony surface
PC-MRV	● No use of contrast materials ● Optimized suppression of stationary background tissues ● Can quantify flow and determine flow direction	◆ Time-consuming (>15 min) ◆ More susceptible to motion artifact ◆ Limited diagnostic value for diagnosing cortical vein thrombosis ◆ Overestimation of hypoplastic sinus	➣ Better background suppression for venous sinuses and large cortical veins ➣ Sensitive for diagnosing CVT ➣ Evaluating pathology in the vessels which are close to the bony surface
CE-MRV	● Unlikely to be affected by complex flow ● Shorter acquisition times ● Produce high-quality angiograms	◆ Gadolinium administration ◆ Not for patients with pregnancy, contrast material allergy or abnormal renal function	➣ Evaluating both the venous sinuses and smaller cortical veins ➣ The reference standard for venous diseases such as CVT
SWI	● No use of contrast materials ● No arterial contamination ● Shorter acquisition times	◆ Cannot guarantee the precise location of measurement ◆ Not accurate when distinguishing between hemorrhage, thrombosis, and calcification lesions ◆ Not suitable for venous thrombosis in chronic phase	➣ Visualization of the anatomy of cerebral venous system ➣ Quantitative measurements of deep cerebral veins ➣ Useful in imaging cortical veins thrombosis ➣ Predicting venous stroke severity ➣ Detecting dural arteriovenous fistula and vascular malformation

We hope that researchers and clinicians can take advantage of the evolving imaging technology, expand their research groups, and deepen their understanding of normal and abnormal cerebral venous and sinus radiography imaging. While continuing to enrich our understanding of the relatively scarce anatomical and morphological imaging data of the cerebral venous system, more imaging diagnostic parameters and evaluation indicators of the corresponding diseases will be established to help solve the problems of missed diagnosis and overdiagnosis in the clinical imaging assessment of cerebral venous system diseases. Meanwhile, imaging assessment should not be limited to physiological parameters, such as hemodynamics. Still, it should broaden the scope of functional imaging of the cerebral venous system to comprehensively characterize the structure and function of the cerebral venous system in both normal and pathological states. In this process, we expect that researchers will use advanced imaging technology to discover and confirm the link between pathophysiological changes in the cerebral venous system, other central system diseases, and systemic diseases.

## Author contributions

LL, XJ, and CZ conceptualized the study. LL acquired and analyzed the data and wrote the original draft. YW and XJ contributed to funding acquisition. KZ, RM, and JD provided critical revisions. LL and CZ contributed to writing – review and editing. All authors have contributed to the manuscript and approved the submitted version.
